# Automated measurement of hydrops ratio from MRI in patients with Ménière’s disease using CNN-based segmentation

**DOI:** 10.1038/s41598-020-63887-8

**Published:** 2020-04-24

**Authors:** Young Sang Cho, Kyeongwon Cho, Chae Jung Park, Myung Jin Chung, Jong Hyuk Kim, Kyunga Kim, Yi-Kyung Kim, Hyung-Jin Kim, Jae-Wook Ko, Baek Hwan Cho, Won-Ho Chung

**Affiliations:** 1 Department of Otorhinolaryngology-Head and Neck Surgery, Samsung Medical Center, Sungkyunkwan University School of Medicine, Seoul, Korea; 2 Medical AI Research Center, Samsung Medical Center, Seoul, Korea; 3 Department of Medical Device Management and Research, SAIHST, Sungkyunkwan University, Seoul, Korea; 4 Department of Radiology, Samsung Medical Center, Sungkyunkwan University School of Medicine, Seoul, Korea; 5 Department of Digital Health, SAIHST, Sungkyunkwan University, Seoul, Korea; 6 Statistics & Data Center, Research Institute for Future Medicine, Samsung Medical Center, Seoul, Korea; 7 Department of Clinical Pharmacology and Therapeutics, Samsung Medical Center, Seoul, Korea

**Keywords:** Inner ear, Magnetic resonance imaging, Neurological disorders

## Abstract

Ménière’s Disease (MD) is difficult to diagnose and evaluate objectively over the course of treatment. Recently, several studies have reported MD diagnoses by MRI-based endolymphatic hydrops (EH) analysis. However, this method is time-consuming and complicated. Therefore, a fast, objective, and accurate evaluation tool is necessary. The purpose of this study was to develop an algorithm that can accurately analyze EH on intravenous (IV) gadolinium (Gd)-enhanced inner-ear MRI using artificial intelligence (AI) with deep learning. In this study, we developed a convolutional neural network (CNN)-based deep-learning model named INHEARIT (INner ear Hydrops Estimation via ARtificial InTelligence) for the automatic segmentation of the cochlea and vestibule, and calculation of the EH ratio in the segmented region. Measurement of the EH ratio was performed manually by a neuro-otologist and neuro-radiologist and by estimation with the INHEARIT model and were highly consistent (intraclass correlation coefficient = 0.971). This is the first study to demonstrate that automated EH ratio measurements are possible, which is important in the current clinical context where the usefulness of IV-Gd inner-ear MRI for MD diagnosis is increasing.

## Introduction

Ménière’s disease (MD) is a multifactorial disorder with typical symptoms of recurrent spontaneous attacks of vertigo, fluctuating hearing loss, tinnitus, and sensations of ear fullness. Endolymphatic hydrops (EH) is a pathological finding where the endolymphatic spaces are distended by enlargements of endolymphatic volume, a histologic hallmark of MD^1–3^. According to a 1995 consensus statement from the Committee on Hearing and Equilibrium of the American Association of Otolaryngology-Head and Neck Surgery (AAO-HNS), “certain” MD cases can only be confirmed by the histological demonstration of EH in postmortem temporal bone specimens^4^. Therefore in 2015, a committee of the Bárány Society revised the diagnostic criteria to remove the concept of “certain MD”^5^. Thus far, the diagnostic criteria have been changed due to the lack of tools to objectively find EH during life. However, with the advancement of imaging technology, MRI can be used to identify endolymphatic hydrops in MD patients as an objective marker.

In 2004, Duan *et al*. succeeded in visualizing EH *in vivo* for the first time in a guinea pig using 4.7 T MRI^6^. Nakashima *et al*. succeeded in confirming EH after injecting contrast media through intratympanic (IT) and intravenous (IV) injections into MD patients using 3 T MRI^7,8^.

Recently, many reports have been published regarding the use of MRI to assess EH. In particular, IV gadolinium (Gd)-enhanced inner-ear MRI has shown good results^9,10^. We have also proven through previous studies that IV-Gd inner-ear MRI is very useful for diagnosing MD by demonstrating the correlation of hydrops with audiovestibular results^11^. IV-Gd inner-ear MRI is less invasive and are much more efficient because it requires less time after the injection of the contrast agent compared to the IT method (4 hr vs. 24 hr) and can evaluate both sides simultaneously^12^. As Gürkov states, although vestibular migraines and MD are not easily differentiated by other methods due to overlapping symptoms, inner-ear MRI can clearly distinguish between them^13^. Therefore, the inner ear diseases associated with endolymphatic hydrops (hydropic inner ear disease) might be differentiated using inner-ear MRI. For this purpose, it is necessary to accurately and consistently calculate the EH ratio. Direct assessment of endolymphatic hydrops using MRI can be applied in clinical practice using the semi-quantification and grading protocol suggested by Naganawa *et al*., which is currently the most widely used^9^. However, to quantify the exact EH ratio, time consuming manual processes were required. HYDROPS (HYbriD of Reversed image Of Positive endolymph signal and negative image of positive perilymph Signal) or HYDROPS-Mi2 (Multiplied with heavily T_2_-weighted MRI cisternography) should be created using a specific brand of image viewer software. Additionally, all cochlear and vestibule boundaries must be drawn manually along the contour on MRI. This manual process is a very time-consuming and cumbersome process and is inefficient for clinical settings. An automated analysis system could be a good option to accurately calculate EH ratios in real time without a time consuming, complicated process. Previous studies have evaluated the automatic segmentation of inner-ear organs. For example, Bouchana *et al*.^14^ described semi-automatic CT image segmentation, which consists of combining thresholding techniques and manual segmentation, but expert intervention is required to localize some points for the segmentation process. Gürkov *et al*.^15^ applied a random forest classifier and a Niblack segmentation algorithm to a 3D-reconstructed image and measured the endolymph and total fluid space. For more automated and semantic segmentation of individual organs (cochlea and vestibule), we investigated deep-learning algorithms. The simplest approach would be to use fully connected artificial neural networks (ANN). However, this would be very computationally expensive because every pixel is linked to every neuron. A convolutional neural network (CNN) solves this issue by filtering the connections by proximity, i.e., each neuron accepts inputs from a subsection (relative receptive field in the image) of the lower layer, making it computationally manageable^16^. In addition, subsection-based processing mimics how individual cortical neurons function (a small portion of a complete visual field), where components of the CNN operate on local input regions. Accordingly, CNNs have demonstrated good performance in semantic segmentation in natural images as well as medical images^17,18^.

**Figure 1:**
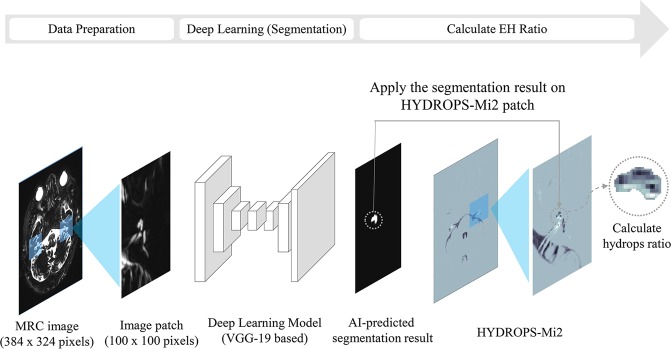
The proposed INHEARIT framework. MRC images (384 ×324 pixels) are cropped into patches (100 × 100 pixels) during the data preparation stage, and the patches are fed into the deep-learning network. The segmentation results are applied to HYDROPS-Mi2 patches as masks, and the endolymphatic hydrops (EH) ratio is calculated from the segmented areas.

In this study, we developed a CNN-based deep-learning model named INHEARIT (INner-ear Hydrops Estimation via ARtificial InTelligence) for the automatic segmentation of the cochlea and vestibule and for the calculation of the EH ratio in the segmented region (Fig. 1). Using our framework, we can estimate the EH ratio accurately and quickly. By analyzing hydrops with MRI, the diagnosis of MD is more accurately made by differentiating from other diseases without hydropic ears presenting hearing loss, tinnitus, ear fullness, and vertigo attacks. In addition, automatic quantitative analysis of hydrops ratios using inner ear MRI may be applied for assessing the stage of disease and prognosis. In this study, we developed an algorithm that can calculate the EH ratio from IV-Gd inner-ear MRI using CNN.

## Results

### Intersection-over-union of AI-prediction and ground truth values

Quantitative results from INHEARIT on the different configurations of the dataset and models are presented in Table 1. A subset of the dataset only includes annotations on the organ of interest (the most obviously visible organ) on the ipsilateral side of each representative slice, which we refer to as a selectively annotated dataset (SA). The rest of the dataset has annotations on both the cochlea and vestibule (regardless of whether it is the organ of interest), which we refer to as the fully annotated dataset (FA). Performances are represented along with average intersection-over-union (IoU) for all classes of 5-fold cross validation. Our deep-learning semantic segmentation model is based on the VGG-19 network architecture designed by the Visual Geometry Group (VGG) from the University of Oxford^19^. The two approaches to feed inputs to our INHEARIT models as a concat3into1VGG network and 3into3VGG network are compared (model descriptions are written in the Materials and Methods section). When we trained both models using SA, the average IoUs were 0.497, 0.533, and 0.528 for low, moderate, and high augmentation, respectively, with the concat3into1VGG and 0.620, 0.716, and 0.711 with the 3into3VGG, respectively. Our results confirmed that 3into3VGG performed better than concat3into1VGG. Therefore, we adopted 3into3VGG for our subsequent experiments.

**Table 1:** Performance of INHEARIT (INner ear Hydrops Estimation via ARtificial InTelligence) trained with the dataset according to the annotation: fully annotated dataset (FA), selectively annotated dataset (SA), and both FA and SA (FASA).

Model	Experiment	Number of Original Patches	Dataset	Augmentation	IoU (Avg ± SD)
concat 3into1VGG	**_**	262	SA	Low	0.497 ± 0.022
				Moderate	**0.533** ± **0.024**
				High	0.528 ± 0.017
3into3VGG	1	110	FA	Low	0.580 ± 0.637
				Moderate	0.646 ± 0.033
				High	**0.701** ± **0.024**
	2	262	SA	Low	0.620 ± 0.026
				Moderate	**0.716** ± **0.018**
				High	0.711 ± 0.037
	3	372	FASA	Low	0.635 ± 0.013
				Moderate	0.705 ± 0.002
				High	**0.706** ± **0.011**

IoU = intersection-over-union; Avg = average; SD = standard deviation; FA = fully annotated dataset; SA = selectively annotated dataset; FASA = both FA and SA.

The two models of concat3into1VGG (three slices were concatenated and entered into a VGG-based network) and 3into3VGG (three slices were independently fed into each of the VGG-based networks) were compared. Numbers in bold indicate the highest performance for each item.

**Figure 2:**
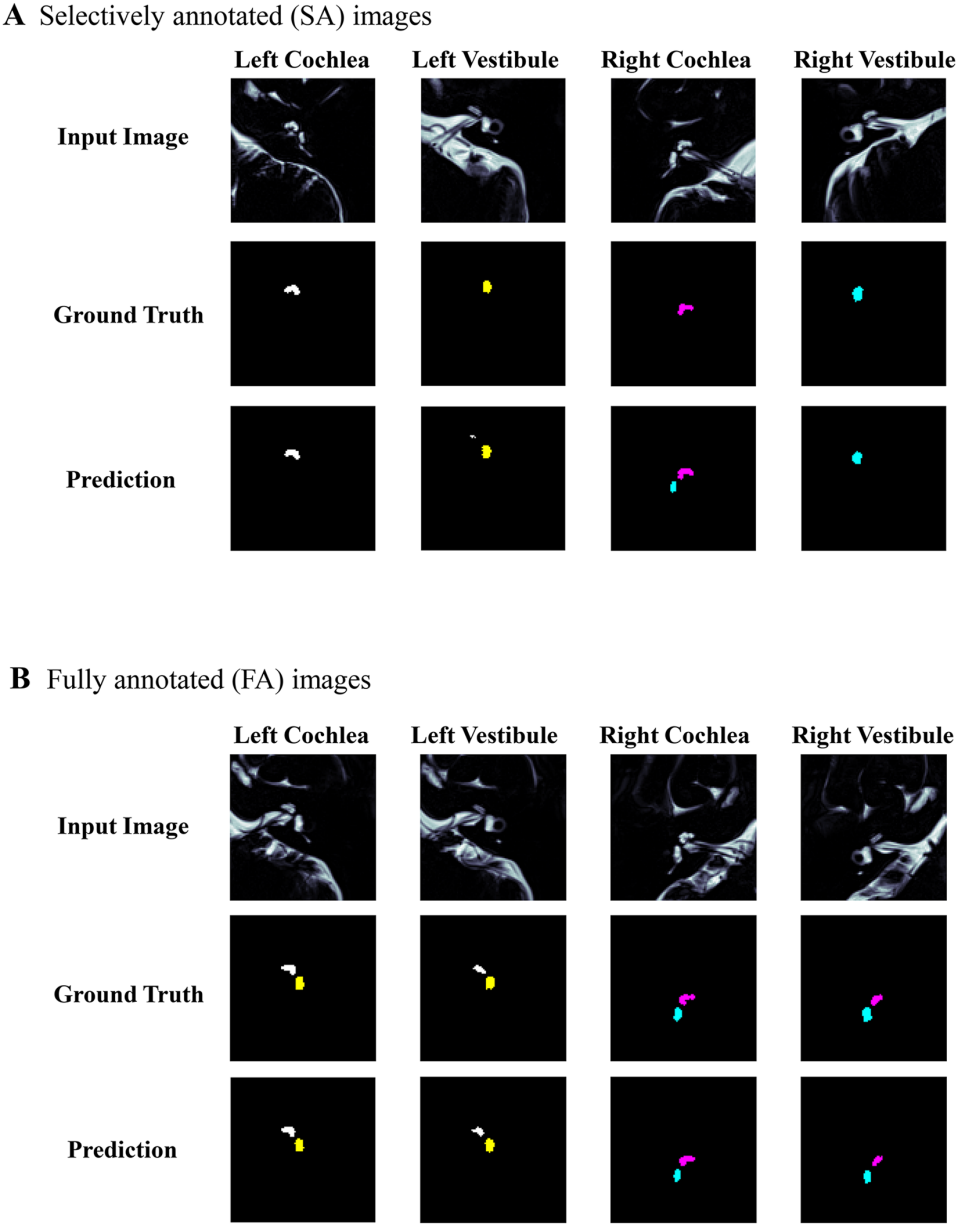
AI-based segmentation results from (**A**) the selectively annotated (SA) dataset and (**B**) the fully annotated (FA) dataset. Those examples show that AI-based prediction performs well compared to physicians’ annotations (ground truth).

Clinician’s annotations on the regions of the cochlea and vestibule using MR cisternography (MRC) images are regarded as the ground truth. The deep-learning segmentation model learns from these annotations together with the input MRC images. Figure 2 shows examples of the ground truth and prediction results from models using FA and SA. It is notable that the trained model predicts not only the most visible organs, but also less-obvious visible organs at the second and third columns in Fig. 2A even though the model was trained with SA. Figure 2B shows the segmentation results with FA where we only calculated IoU for the organs of interest.

The model trained with SA (Experiment 2 in Table 1) exhibited the highest values in average IoU in all the experiments, as shown in Table 1. We assumed that the training models with data scheduling from simple to complex datasets can improve learning efficiency. Therefore, we further fine-tuned the SA-based models to improve their performance. In Table 2, Experiments 4 and 5 fine-tuned the model with FA and FASA, respectively, and Experiments 6 and 7 further fine-tuned the model with only the organs of interest according to FA and FASA, which we named FA’ and FA’SA. In all the experiments shown in Table 2, the validation dataset was FASA, which did not overlap with the training dataset. Experiments 4 and 5 showed improved average segmentation results (IoU) at all augmentation scales compared with the original SA-based models. Fine-tuning with FASA (Experiment 5 in Table 2) outperformed FA (Experiment 4 in Table 2) in all augmentations. Greater augmentation scales in the fine-tuning stages yielded higher IoU values, indicating more precise segmentations. Experiments 6 and 7 in Table 2 showed either improvements or deteriorations in performance. The models in Experiment 6 in Table 2 were fine-tuned with FA’, which included only obviously visible organs, and the segmentation performances were increased to moderate/high augmentation. In Experiment 7 in Table 2, the fine-tuned models using FA’SA yielded decreased IoU values at moderate/high augmentation. From the experiments, we discovered that the fine-tuned model (SA → FASA) exhibited better average IoU values (0.761) than the model trained with the FASA dataset from scratch (0.706 in the last row of Table 1).

**Table 2:** Performance of INHEARIT fine-tuned with item 2 (SA, moderate augmentation) with various dataset combinations.

Experiment	Number of Original Patches	Dataset	Augmentation	IoU (Avg ± SD)	IoU_(2)_^a^
4	110	SA → FA	Low	0.686 ± 0.026	+
			Moderate	0.724 ± 0.030	+
			High	**0.749** ± **0.013**	+
5	372	SA → FASA	Low	0.702 ± 0.032	+
			Moderate	0.760 ± 0.014	+
			High	**0.761** ± **0.036**	+
6	60	SA → FA’	Low	0.610 ± 0.040	−
			Moderate	**0.743** ± **0.031**	+
			High	0.716 ± 0.027	+
7	322	SA → FA’SA	Low	0.642 ± 0.025	+
			Moderate	**0.678** ± **0.018**	−
			High	0.674 ± 0.016	−

IoU = intersection-over-union; Avg = average; SD = standard deviation; SA = selectively annotated dataset; FA = fully annotated dataset; FA’ = main organs only in FA, FASA = both SA and FA, FA’SA = main organs only in FASA; IoU = intersection-over-union.

^a^Loss (−) or gain (+) in IoU compared with Experiment 2 in Table 1 at the same augmentation scale.

Numbers in bold indicate the highest performance for each item.

### Agreement analysis of the endolymphatic hydrops ratio via intraclass correlation coefficients

**Figure 3:**
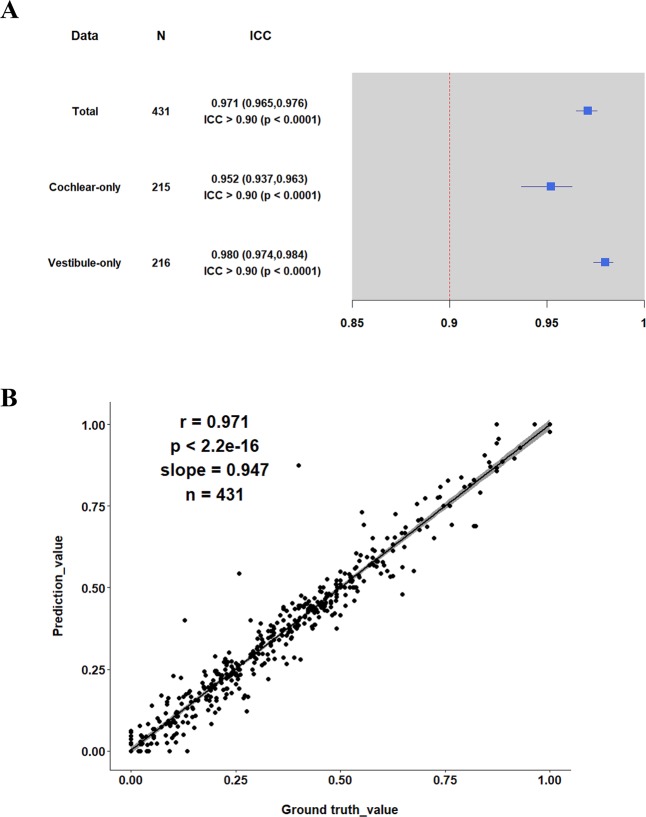
Agreement analysis of the endolymphatic hydrops ratio via intraclass correlation coefficient (ICC), showing (**A**) ICC mean values (maximum and minimum) and p-values for the overall, cochlea-only, and vestibule-only and (**B**) scatter plots for all ICCs between the ground truth and prediction by INHEARIT network values.

**Figure 4:**
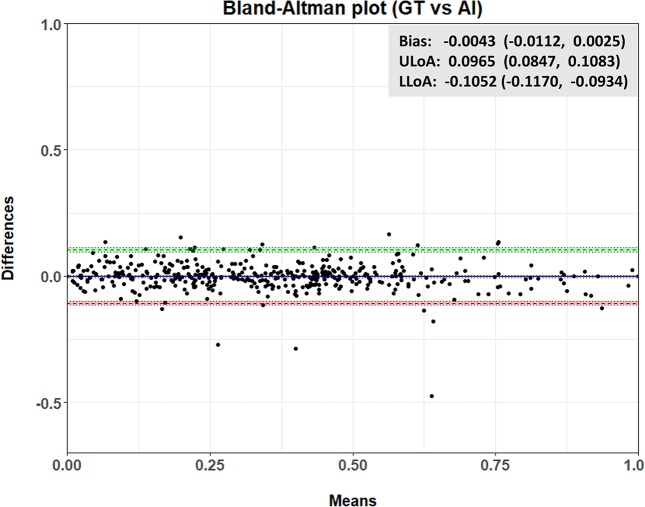
Bland-Altman plot for the total (cochlea and vestibule) dataset. The green line indicates the upper limit of agreement (ULoA), while the red line indicates the lower limit of agreement (LLoA).

In all the fine-tuned experiments in Table 2, the segmentation performance of FASA (Experiment 5 in Table 2) was best for training the INHEARIT model at all augmentation scales. Therefore, we analyzed the EH ratio agreement between the physician-calculated ratio and the ratio predicted by INHEARIT. The average interclass correlation coefficient (ICC) value for an entire image was 0.971, while the average ICC of the vestibule images (0.980) was higher than the cochlea images (0.952) (Fig. 3A). Scatter plots (Fig. 3B) show that the cochlea and vestibule had a good agreement between the ground truth value and the prediction value. The results of the Bland-Altman plot in Fig. 4 also showed that the differences between the ground truth and prediction are very small and high in agreement. The average EH ratio calculation time of an organ was 0.168 seconds.

## Discussion

Using our proposed INHEARIT framework, we obtained high segmentation results up to 0.761 for IoU, with an ICC of 0.971 between the expert physicians and AI. Once INHEARIT was connected to the PACS system, new patient EH ratios were analyzed within 1 second. This increased the speed of the EH ratio measurement compared with current calculation results measured manually using special programs (e.g., OsiriX MD). In current clinical practice, EH ratio measurements require approximately 15–20 minutes to extract and copy one MRI, create a HYDROPS-Mi2 image, draw the ROI, and calculate and report the ratio. In addition, it is difficult for the human eye to distinguish in detail each turn of the cochlea and utricle or saccule from the vestibule. We also developed an automatic calculation process in the Python environment that mimicked the manual calculations performed in the clinic. This enabled the EH ratio to be calculated very rapidly. With our suggested protocol, the EH ratio can be easily read and diagnosed even in institutions with no expert physicians to interpret inner-ear MRI.

Although there is no accurate numeric diagnostic criterion for EH ratios in MD, true-positive versus false-positive rates could not be calculated, but the matching degree we achieved was very high. Direct EH visualization is important during clinical diagnosis not only for MD, but also for many other diseases. For example, detecting EH in patients with recurrent low-frequency hearing loss or with nonspecific dizziness could be helpful^20^.

In developing INHEARIT, fully convolutional layers (VGG-19) were used to extract cochlea and vestibule features. Conventional CNN models that were successfully applied to natural images may not be able to fully represent the characteristics of grey-scale MRI. Therefore, we developed a model appropriate for IV-Gd inner ear MRI not only by fine tuning additional deconvolution layers, but also by manipulating multiple networks. We confirmed that the parallel three-network configuration outperformed the one-network configuration, even when using three consecutive images as input (Table 1). We believe that each network of the parallel configuration could extract target and auxiliary features more effectively with our automated algorithm. It is worth exploring whether other network architectures, such as U-Net^21^, could perform segmentation, but our VGG-based models have already satisfied the criteria for clinical use^22^. Therefore, we did not evaluate other network architectures. Nevertheless, further evaluation of other network architectures or optimization methods will be helpful for better clinical use.

We obtained highly satisfactory results, especially considering that we only had 124 image stacks. There are several reasons why good results were achieved from our small training dataset. First, the size and location of the inner ear organs were similar regardless of age or gender^23^ knowing that these organs do not grow or change shape after birth^19,24^. Second, we utilized curriculum learning, which applies simple learning concepts first and then gradually introduces more complex concepts^25^. We then organized the schedule so that learning started from the obviously visible organ dataset (SA) and then, to improve performance, was fine-tuned with exposure to the less-obviously visible dataset. Lastly, dataset augmentation was performed to compensate for low data quantity. We applied flipping, intensity changing, and random shift cropping of the original images to amplify the training dataset.

As shown in Table 1, the model trained only with SA exhibited a better performance than the one with FA. As mentioned above, FA annotated organs of interest as well as less visible organs in the same slice. The less obviously visible organ annotations in FA were widely variable by shape. As a result, this may have been confusing for our model given the limited amount of training data. We interpret this to mean that high segmentation performance can be achieved when only main organs of interest are used in training from baseline. That segmentation with SA showed comparable performance with FASA despite its smaller dataset size for training supports this idea.

Regarding the effectiveness of augmentation in deep-learning performance, the model trained with the FA dataset (Experiments 1, 3, 4, and 5 in Tables 1 and 2) exhibited improved performance by augmentation size, whereas models trained with organs of interest from the SA or FA’ dataset (Experiment 2, 6, and 7 in Tables 1 and 2) showed poorer performance at moderate-high augmentation. Therefore, we assume that augmentation is more effective when there are greater shape variations in image annotation in the training dataset. The FASA (Experiment 3 in Table 1) yielded performance increases through augmentation at all scales, which is consistent with the FA results.

It is notable that training scheduling using the curriculum learning concept worked well in this study. Compared with the results in Experiment 3 of Table 1 (training both simple and complex concepts from scratch), the results in Experiments 4 and 5 of Table 2 (training with simple concepts first and then fine-tuning with complex concepts) yielded improved performances. Furthermore, when fine-tuning, it was more effective to use both complex and simple data (Experiment 5) rather than only complex data (Experiment 4). This fit expectations that Experiments 4 and 5 yielded better performances than Experiments 6 and 7 because the latter experiments only used simple data for fine-tuning, even though the validation dataset included both simple and complex data.

Our research has several limitations. First, the number of images used for training and validation was relatively small compared to other deep-learning studies. To overcome this limitation, we used data augmentation at various scales. Second, image analysis did not include a control group with no MD-associated symptoms. Therefore, an additional study is being performed with healthy participants who exhibit no dizziness or hearing loss. Third, full stack image validation was not applied in this study. The developed model used manually pre-defined representative image slices that included organs of interest. Future studies using full stack analysis will advance our model into a fully automated framework. Lastly, our study included images produced with a single MRI instrument (Siemens) because the suggested sequence for analysis of the EH ratio is specialized for this device. Therefore, we did not perform external validations. However, future external validations will be helpful to confirm the value of our INHEARIT system for clinical use once the sequence is available on other MRI machines.

## Conclusions

We demonstrated that IV-Gd inner-ear MRI analysis using deep learning is fast and accurate. If MRI can be combined with image analysis using deep learning, inner-ear MRI will be a useful objective diagnostic tool. In addition, our INHEARIT system is practical for broad usage to assess and diagnose any disease associated with endolymphatic hydrops.

## Materials and methods

### Subject enrollment

MRI data from 124 subjects (57 males, 67 females; mean age = 49.3 yr, Age range = 17~76 yr) were evaluated for this study. All subjects underwent IV-Gd inner-ear MRI and pure-tone audiometry (PTA) at an outpatient clinic. Of the total 124 images, 83 were diagnosed with definite MD (unilateral or bilateral) according to the revised diagnostic criteria from the 2015 Classification Committee of the Bárány Society^5^. Eleven patients were diagnosed with migraine-associated dizziness, 7 with vestibular neuritis, and the remaining 23 with probable MD.

Patients who underwent surgical treatment or intratympanic gentamicin treatment for intractable vertigo were excluded from this study. Written informed consent was obtained from all participants prior to conducting the study. This study was approved by the Institutional Review Board of Samsung Medical Center following the tenets of the Declaration of Helsinki (IRB File No. 2018-11-020-003).

### Intervention

The MRI protocol below is the same as the one originally reported by Naganawa *et al*. in 2012^26^. IV-Gd inner-ear MRI was performed on a 3.0-T unit (MAGNETOM Skyra; Siemens Medical Solutions, Erlangen, Germany) using a 32-channel array head coil. All patients waited 4 hours after a single dose (0.1 mL/kg or 0.1 mmol/kg body weight) of IV-administered gadobutrol (gadolinium-DO3A-butriol, GADOVIST 1.0; Schering, Berlin, Germany) before undergoing MRI. All patients underwent heavily T2-weighted (hT2W) MR cisternography for the anatomical reference of total endolymphatic fluid, hT2W– 3D-FLAIR with an inversion time of 2250 ms (positive perilymph image, PPI), and hT2W–3D-IR with an inversion time of 2050 ms (positive endolymph image, PEI) for evaluating endolymphatic hydrops. Repetition time was 9000 ms, echo time was 540 ms, and voxel size was 0.5 * 0.5 * 1.0 mm.

The PEI parameters were the same as for PPI, except that PEI had an inversion time of 2050 ms. MR cisternography (MRC), PPI, and PEI employed identical field of views, matrix sizes, and slice thicknesses to facilitate comparisons. We produced HYDROPS images on the scanner console by subtracting the PEI from the PPI. To increase the contrast-to-noise ratio of the HYDROPS images, HYDROPS-Mi2 images were generated on a DICOM viewer (OsiriX MD image software, version 7.5.1 64-bit; Pixmeo Sarl, Bernex, Switzerland, https://www.osirix-viewer.com) by multiplying the HYDROPS and MRC images^9^.

All the patients underwent PTA at 6 frequencies (0.25, 0.5, 1.0, 2.0, 4.0, and 8.0 kHz). We used a semi-automated testing device in a sound-attenuating booth that met the prevailing standards for maximum permissible ambient noise levels during audiometry (ANSI, 1977).

### Data annotation by physicians

One neuro-radiologist and one neuro-otologist independently evaluated MRI. According to the methods proposed by Naganawa *et al*.^9^, each physician manually drew a contour of the cochlea and vestibule on the MRC image, which is the region of interest (ROI). Setting the ROI occurred as follows: (1) Before drawing the contour of the cochlea or vestibule margin on the image, the image window level and width was altered to 400/1000 to obtain the best visual clarity. (2) For the cochlea ROI, the slice visualizing the cochlea turns (basal, middle, and apical) was selected. If every turn was visible on 2 or more slices, the slice with the largest height of the modiolus was chosen as a representative cochlea slice. (3) For the vestibular ROI, the lowest slice where the lateral semicircular canal (LSCC) ring was visible for more than 240° was selected as a representative vestibular slice and the ampulla was excluded when drawing the ROI for the vestibule on MRC images. ROIs drawn on IV-GD were copied and pasted onto HYDROPS-Mi2 images. The histogram function in the OsiriX program was then used to estimate the numbers of pixels in the ROI and the numbers of pixels with negative signal intensity values (i.e., endolymph) in the ROI. The EH ratio was then manually calculated as the number of pixels for the endolymph in the ROI divided by the total number of pixels in the ROI.

### Data preparation for deep learning

We generated annotation masks (ground truth for deep learning) for each of the left cochlea (LC), left vestibule (LV), right cochlea (RC), and right vestibule (RV) classes by filling the regions inside the annotated ROIs. The areas of the cochleae and vestibules were relatively small according to the original whole MRC images (384 ×324 pixels). Therefore, we cropped 100×100-pixel windows from each side of the inner-ear images at the left [215,238] and right [204,92] reference points with the cropping reference points being determined by a radiologist. All the ROIs for the entire dataset resided inside the cropped windows.

We performed data augmentation to increase the number of training images for deep learning given the limited number of original image data points. Flip, random shift cropping, and brightness control were applied for data augmentation. Since the cochleae and vestibules exhibited symmetric characteristics in the MRC images, we flipped the MRC image patches together with the corresponding annotations. Random shift cropping was performed on the images within a range of 8 pixels around the reference points in the up, down, left, right, and diagonal directions (total states: 9). Brightness changes to the images were applied in the range from −50 to 50 of pixel intensity with a variation step of 1 or 10 depending on the augmentation degree. We attempted three types of augmentations: low augmentation by flipping and random shifting (144 times); moderate augmentation by flipping, random shifting, and 10 steps of brightness change (1,584 times); and high augmentation by flipping, shifting, and one step of brightness change (14,544 times).

### INHEARIT model training

**Figure 5:**
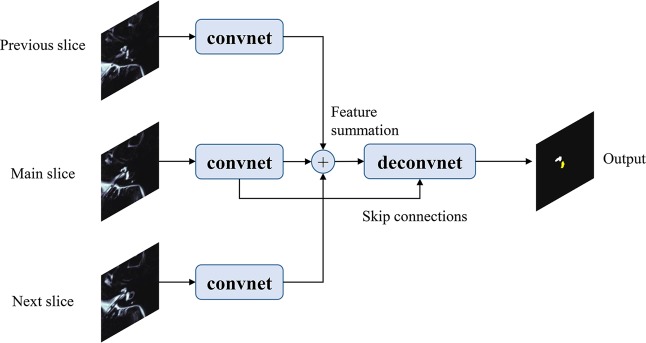
Concept of the *3into3VGG* of the INHEARIT network. The network received three independent MRC images into each convolutional network (*convnet*), and features from the three layers are summated before the deconvolutional layers. Two skip connections from the main convolutional network are connected to the deconvolutional network (*deconvenet*).

The VGG network is comprised of 16 convolution layers and three fully connected layers trained for natural image classification. The INHEARIT consists of three VGG networks (each network uses separate image slices as input), which adopted the front part (up to the 15th convolutional layer) of the VGG-19 layer and connected three convolution layers and another three deconvolution layers for organ segmentation. We fed three consecutive MRC images to the INHEARIT, centering at the slice of interest. The main slice of interest and its previous and next slices were loaded together from the full MRI stack. We attempted two different approaches to feed inputs into the system. First, three slices were concatenated and entered into a VGG-based network as a 3-channel input image to extract the features (a.k.a. concat3into1VGG). Second, three slices were independently fed into each of the VGG-based networks. The feature maps after addition of the three convolutional layers (before the deconvolutional layers) were summated over the three networks (a.k.a. 3into3VGG, Fig. 5). Afterwards, for both approaches, the maps were up-sampled through deconvolution layers and finally generated a prediction output image of 100 ×100 pixels, the same size as the input image. Model parameters were optimized using the Adam optimizer with a learning rate of 1e-6, dropout of 0.6, and batch size of 4, which were manually tuned with a grid search. The model was trained on graphical processing units (GPUs; NVIDIA GTX 1080Ti).

### Training with fully annotated and selectively annotated datasets

We performed experiments on the FA, SA, and both datasets (FASA) separately. We also attempted to adopt a transfer learning scheme, which transfers features trained from one domain to another domain to improve learning performance^27^. To apply 5-fold cross validation, the patches in the dataset were randomly divided into 5 equal-sized partitions so that 80% and 20% of the dataset could be applied to training and validation, respectively, wherein a single partition was retained for model validation and the other 4 partitions were used as training data. This process was repeated 5 times, each time using a different partition as validation data. For quantitative comparisons between the ground truth and the predicted segmentation results, we measured IoU in each training epoch to determine regional overlap.

$${\rm{Intersection}}-{\rm{over}}-{\rm{union}}\,({\rm{IoU}})=\frac{{\rm{Area}}\,{\rm{of}}\,{\rm{overlap}}}{{\rm{Area}}\,{\rm{of}}\,{\rm{union}}}$$

### EH ratio calculation

To obtain automated measurements of EH ratios from the segmented results, we developed an algorithm to calculate the EH ratio. Our INHEARIT method covers automatically generated HYDROPS-Mi2 images by multiplying HYDROPS and MRC images and calculating endolymphatic EH ratios from the deep-learning-based segmented area. The EH ratio is defined as follows:

$${\rm{EH}}\,{\rm{Ratio}}=\frac{{\rm{Total}}\,{\rm{number}}\,{\rm{of}}\,{\rm{pixels}}\,{\rm{with}}\,{\rm{negative}}\,{\rm{value}}\,{\rm{in}}\,{\rm{the}}\,{\rm{segmentation}}\,{\rm{area}}}{{\rm{Total}}\,{\rm{number}}\,{\rm{of}}\,{\rm{pixels}}\,{\rm{in}}\,{\rm{the}}\,{\rm{segmentation}}\,{\rm{area}}}$$

Negative value represents endolymphatic space (non-enhanced fluid) except for perilymph which was enhanced by a Gd. EH ratios were calculated using both ground truth and prediction results. Correlation coefficients between the two ratios were then computed.

### Statistical analysis

We investigated the agreement between the ground truth values calculated by physicians and the AI-based predicted values using the single-score intraclass correlation coefficient based on a two-way model, Pearson’s correlation coefficient, and the Bland-Altman plot. Analyses were performed using R core team (2019)^28^.
